# Effect Modification of Alcohol Use on Epilepsy: NHIS Longitudinal Study

**DOI:** 10.3390/biomedicines14051001

**Published:** 2026-04-28

**Authors:** Sri Banerjee, W. Sumner Davis, Kay Banerjee, Joseph McMillan, Claret Onukogu, Pat Dunn, Arturo Olazabal, Mekuria Asfaw, Heather Esnaola, Stephanie Watkins, Rafael Gonzales-Lagos

**Affiliations:** 1 College of Health Sciences and Public Policy, Walden University, Minneapolis, MN 55401, USA; srikanta.banerjee2@mail.waldenu.edu (S.B.); dr.sumner.davis@gmail.com (W.S.D.); karen.banerjee@waldenu.edu (K.B.); joseph.mcmillan@mail.waldenu.edu (J.M.); claret.onukogu@waldenu.edu (C.O.); arturo.olazabal@waldenu.edu (A.O.); mekuria.asfaw@waldenu.edu (M.A.); heather.esnaola@waldenu.edu (H.E.); stephanie.watkins5@waldenu.edu (S.W.); boranda131@icloud.com (R.G.-L.); 2 Department of Counseling, Keystone Human Services, Harrisburg, PA 17110, USA; 3 Lakeview Specialty Hospital and Rehabilitation, Waterford, WI 53185, USA; 4 American Heart Association, Digital Solutions, Leander, TX 75231, USA

**Keywords:** epilepsy, alcohol abuse, alcohol withdrawal seizures, comorbidity, antiseizure medication, neurobiology

## Abstract

**Introduction:** The relationship between epilepsy and alcohol use is complex and clinically significant. Alcohol acts as a neurochemical modulator capable of lowering the seizure threshold during both intoxication and withdrawal, while chronic misuse may contribute to epileptogenesis through neuronal injury, metabolic stress, and neurotransmitter dysregulation. However, the long-term impact of alcohol use on mortality among people with epilepsy (PWE) remains insufficiently characterized. The purpose of this study was to assess all-cause mortality risk among individuals with epilepsy based on alcohol use history, stratified by race/ethnicity. **Methods:** Data from the 2008–2018 National Health Interview Survey (NHIS) were linked to mortality outcomes on 31 December 2019 from the National Death Index (NDI) for U.S. adults aged 18 years and older. PWE and alcohol use were determined using self-reported data. Survival probabilities were estimated using weighted Kaplan–Meier methods, and hazard ratios were calculated using Cox proportional hazards models adjusted for demographic and clinical covariates. **Results:** Our results indicated that among PWE, alcohol use was associated with increased all-cause mortality. The unadjusted hazard ratio (HR) for alcohol use among individuals with epilepsy was 1.30, increasing to 1.40 after multivariable adjustment. In contrast, alcohol use alone without epilepsy was not associated with elevated mortality risk after adjustment. When stratified by race, the combined effect of epilepsy and alcohol use was significantly associated with increased mortality among Black individuals but not White individuals. **Conclusions**: In this nationally representative cohort, the combined presence of epilepsy and alcohol use was associated with higher all-cause mortality compared with alcohol use alone. Racial differences were observed, underscoring the need for integrated clinical care and further research into genetic, biological, and social determinants influencing epilepsy outcomes.

## 1. Introduction

Epilepsy, as defined by the World Health Organization, is a chronic neurological disorder characterized by recurrent, unprovoked seizures resulting from abnormal electrical activity in the brain [1]. This involves various biomolecular, structural, and functional abnormalities, most commonly hippocampal sclerosis and focal cortical dysplasia [1,2]. In recent years, newer seizure animal models have been using light-gated ion channels that turn on cell firing [1,3]. Experiments show that as many as 21 ILAE (International League Against Epilepsy)-recommended genes may represent an “ideal” core set likely able to provide the highest diagnostic efficiency and cost-effectiveness and facilitate gene prioritization when testing patients with whole-exome/whole-genome sequencing [4,5]. This was discovered through a genome-wide analysis of nearly 45,000 people, with DNA from 15,212 individuals with epilepsy and 29,677 without epilepsy [4,5]. There are additional studies that are connecting genetic blueprints to cellular functions and disease states, often at the single-cell level for deeper insights. For example, in a multi-omic sequencing study, authors found, using seizure-prone and seizure-resistant mice, that the mice in two groups had differential susceptibility to withdrawal phenotypes [5]. This was found in F2 mice as well, through genomic, genetic, and transcriptomic analysis [6,7]. Also, withdrawal convulsions were identified as genes that control potassium channels and perturbations of the genetic expression of genomic transformation. Although moderate alcohol use rarely precipitates seizures, heavy consumption and withdrawal are well-established seizure triggers [8]. The connection between epilepsy and alcohol is bidirectional: alcohol can provoke seizures, and people with epilepsy may develop problematic drinking behaviors due to psychosocial stressors and medication effects [9,10,11,12,13].

Epidemiological evidence indicates that alcohol misuse is associated with an elevated risk of seizures and epilepsy, though causality remains debated. A meta-analysis by Woo et al. found that individuals with heavy alcohol consumption had a 2.5-fold increased risk of unprovoked seizures compared with abstainers [11,12]. Moreover, according to this meta-analysis, researchers evaluated both cohort and case–control studies examining alcohol consumption and epilepsy risk. The pooled results showed an increased risk of epilepsy among alcohol users, with a significant dose–response relationship evident in case–control studies [12]. In contrast, cohort studies did not demonstrate a statistically significant association. The findings suggest that heavy alcohol consumption may increase epilepsy risk, while emphasizing the need for additional large-scale cohort studies to clarify causality and define risk thresholds. Similarly, a Polish prison cohort demonstrated a fourfold higher prevalence of epilepsy among those with alcohol dependence [12,13,14]. Conversely, longitudinal studies show inconsistent results, suggesting that confounders such as traumatic brain injury, hepatic dysfunction, or cerebrovascular disease may mediate part of this relationship [13,14]. Nonetheless, population-based data reveal higher epilepsy-related mortality and sudden unexpected death in epilepsy (SUDEP) among individuals with concurrent alcohol misuse [12,14].

What is known are the neurobiological mechanisms, epidemiological patterns, and management strategies concerning the overlap between epilepsy and alcohol abuse [15]. There are multiple meta-analyses and small sample studies assessing the epidemiological patterns [12,13,14,15]. However, what is not known is how alcohol abuse interacts with the relationship between alcohol use and all-cause mortality. Furthermore, our goal is to summarize current scientific understanding and propose directions for integrated clinical care and future research.

## 2. Materials and Methods

The National Health Interview Survey (NHIS) is an annual, nationally representative survey of the U.S. civilian, non-institutionalized population conducted by the National Center for Health Statistics (NCHS). Its primary purpose is to collect detailed information on individual- and household-level sociodemographic and health characteristics. NCHS has developed public-use versions of the NHIS that are linked to death certificate records from the National Death Index (NDI). For this study, we used the 2008–2018 public-use linked mortality files—derived from the 2008, 2010, 2013, 2015, and 2017 NHIS Sample Adult components—which provide mortality follow-up from the date of survey participation through 31 December 2019.

Each potential NDI match is assigned a probabilistic match score, calculated as the sum of the weights assigned to identify data items used in the linkage process. These weights reflect the degree of agreement between information on the NHIS submission record and the corresponding NDI death record. NHIS data for this analysis were obtained through the Integrated Public Use Microdata Series (IPUMS) at the Minnesota Population Center. IPUMS NHIS provides harmonized and recoded versions of the original CDC/NCHS data to facilitate the study of individuals within the context of families and household co-residents.

### 2.1. All-Cause Mortality

We assessed the increased risk of all-cause mortality using hazard ratios derived from the International Classification of Diseases, 10th Revision (ICD-10). Follow-up time for decedents was calculated as the number of months between the month and year of the NHIS interview and the month and year of death. Because the NHIS–NDI linked mortality files report only the quarter of death, we assumed that deaths occurred at the midpoint of the reported quarter (February, May, August, or November).

### 2.2. Alcohol Use

Physical conditions may be exacerbated by alcohol use. In this study, alcohol use was categorized as never used, former user, or current user, and dichotomized into “never used” versus “former user” and “current user.”

### 2.3. Persons with Epilepsy (PWE)

The NHIS epilepsy module we used to select our sample is administered biennially. The key question determining PWE status was: “Have you ever been told by a doctor or other health professional that you have a seizure disorder or epilepsy?” [15]. Our research builds on previous studies by employing a hypothesis-driven methodology, analyzing multi-year data, and applying predictive multivariable models.

### 2.4. Covariates

Our independent variables include various health indicators—such as a history of myocardial infarction and stroke, obesity (yes/no), and chronic kidney disease (CKD)—along with social factors affecting healthcare access and usage. These social determinants encompass poverty status (<200% of the Federal Poverty Level), educational attainment (no high school diploma, high school graduate, some college, college graduate, graduate school), race/ethnicity (non-Hispanic White, non-Hispanic Black, Hispanic, other), age (years), smoking status (current, former, never), and sex/gender (female vs. male).

Unweighted descriptive statistics for respondents with epilepsy were calculated and are presented in Table 1. Demographic variables were weighted using the NHIS-provided sample weights to approximate the U.S. population distribution and to account for oversampling, unequal selection probabilities, and non-response. The normality of continuous variables was assessed using the Shapiro–Wilk test. Categorical variables were summarized as percentages and compared using chi-square tests.

#### Statistical Analysis

To evaluate the association between epilepsy, alcohol use, and all-cause mortality, we estimated complex-sample multiple Cox proportional hazards models adjusting for all covariates. Prior to fitting the Cox regression models, we initially incorporated tests based on Schoenfeld residuals to assess the proportional hazards assumption. The findings indicated that both insurance status and marital status failed to meet the PH assumption in multiple models. Statistical analyses were conducted using SAS (version 9.3; SAS Institute Inc., Cary, NC, USA) and SUDAAN (version 9.0; Research Triangle Institute, Research Triangle Park, NC, USA). In addition, to check the robustness of the association, a series of sensitivity analyses was performed on the available data. Statistical significance was defined as *p* < 0.05.

## 3. Results

In the initial sample of 81,750, there were 1430 individuals who were identified as having epilepsy. After the follow-up period, 6070 cases of mortality were identified. Among the 1430 PWE, 140 individuals experienced mortality. Table 1 provides a detailed account of the demographic characteristics of the participants categorized by a healthcare provider-diagnosed history of epilepsy, utilizing bivariate analysis. The prevalence of epilepsy within the adult population of the United States, specifically among those aged 18 years and older, was recorded at 1.8%. Furthermore, the average age of participants diagnosed with epilepsy was 47.5 years, compared to 48.3 years for those without epilepsy. A statistically significant association (*p* < 0.05) was observed between epilepsy and various conditions, including myocardial infarction, stroke, chronic kidney disease (CKD), obesity, age, race/ethnicity, education, poverty income ratio (PIR), and smoking status. Additionally, a statistically significant correlation was found between epilepsy status and all-cause mortality.

**Figure 1:**
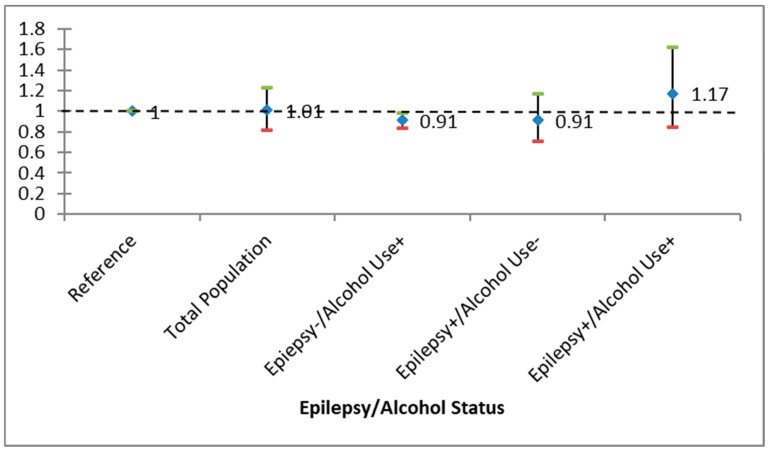
Crude and adjusted hazard ratio (HR) of epilepsy stratified by alcohol use among White Americans. None of the categories were significant, so there is no effect modification. Bars represent 95% confidence intervals for the HR.

**Figure 2:**
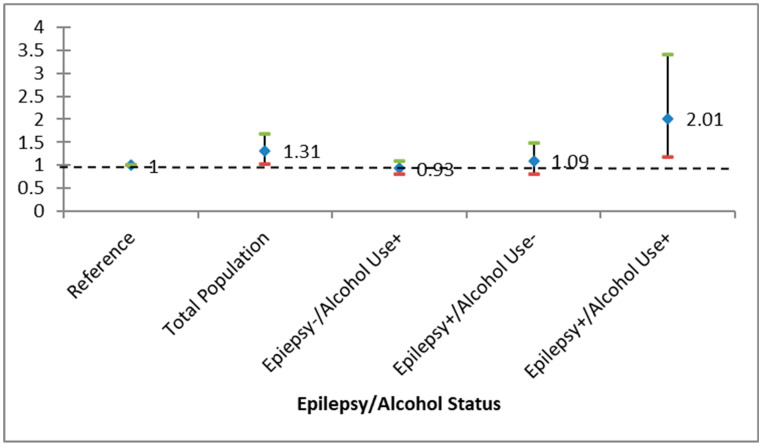
Crude and adjusted hazard ratio (HR) of epilepsy stratified by alcohol use among Black Americans. (Epilepsy+ and Alcohol use+ were the only measures that were *p* < 0.05, demonstrating effect modification.) Bars represent 95% confidence intervals for the HR.

In terms of all-cause mortality, the crude hazard ratio (HR) for PWE among Black individuals was found to be 1.42 (95% confidence interval [CI], 1.06–1.91, *p* = 0.02). Following adjustments for various factors, the HR significantly increased to 2.01 (CI, 1.18–3.40, *p* < 0.01) for PWE who also engaged in alcohol use; however, for those with only alcohol use or epilepsy, the HR did not show a significant elevation. Conversely, regarding overall mortality, the crude hazard ratio (HR) for PWE among White individuals was recorded at 0.97 (95% confidence interval [CI], 0.79–1.18, *p* = 0.73). Even after adjusting for multiple variables, the HR did not demonstrate a significant increase among PWE with alcohol use, nor did it among those with solely alcohol use or epilepsy. As illustrated in Figure 1 and Figure 2, PWE exhibited heightened mortality rates, but only among African Americans. Notably, the survival rate for the PWE group declined more swiftly than that of other groups across both racial categories over time. Table 2 and Table 3 further indicate a greater likelihood of all-cause mortality over time among individuals with epilepsy and alcohol use, with this trend being more pronounced in Black individuals compared to their White counterparts.

## 4. Discussion

In this large, nationally representative, diverse cohort, we found that there may be a direct connection between epilepsy and all-cause mortality. Previous researchers, using small-scale primary studies in epilepsy clinics, have also assessed the deleterious effects of alcohol in PWE [15,16,17,18,19]. For instance, in a systematic review and meta-analysis that synthesized data comprising 27 epidemiological studies involving over 565,000 persons with epilepsy (PWE), researchers found that most psychiatric disorders were significantly more prevalent in PWE [16,17]. These included depression, anxiety, bipolar disorder, psychotic disorders, schizophrenia, obsessive–compulsive disorder, posttraumatic stress disorder, substance use disorders, autism spectrum disorder, and attention-deficit/hyperactivity disorder. Autism spectrum disorder showed the highest relative increase. These findings highlight the substantial psychiatric burden associated with epilepsy and underscore the importance of systematic psychiatric screening and integrated mental health care in epilepsy management [16,17]. Researchers also found that heavy alcohol intake disrupts sleep architecture, causing impaired adherence to antiepileptic medication.

Our epidemiological findings also have certain biological correlates. Previous research has shown that by using maximum electroshock seizure models, the anticonvulsant activity of semicarbazones often involves interaction with voltage-gated sodium ion channels or modulation of GABA receptors in the brain. This activity is linked with its protective index, higher than that for carbamazepine, phenytoin, and valproic acid, influencing the binding mainly due to its hydrogen bonding [15]. This experiment was conducted on a rodent population with the delivery of electrical current (60 Hz alternating current) for a short duration (0.2 s) via corneal or other electrodes [18]. In addition, epileptogenesis triggers molecular changes in the hippocampus, including altered neurogenesis and enhanced expression of neurotrophic factors and proteins [17]. The activation of immune cells like microglia and astrocytes releases pro-inflammatory cytokines that increase neuronal excitability. These inflammatory signals can also compromise the blood–brain barrier.

Furthermore, researchers have found that alcohol dangerously interacts with epilepsy both through pharmacodynamics and pharmacokinetics. This, in turn, lowers the seizure threshold. Pharmacokinetically, since seizure drug levels are metabolized in the liver, this worsens side effects from seizure medications. More specifically, drugs like carbamazepine and alcohol lead to worsening side effects and overall central nervous system (CNS) function [18,19,20,21,22]. Consumption of alcohol may result in breakthrough seizures and withdrawal seizures. Therefore, the recommendation is to practice complete abstinence, as this interrupts seizure control. In addition, pharmacokinetic interactions (e.g., carbamazepine inducing enzymes, valproate inhibiting them) alter drug levels, while pharmacodynamic interactions can cause combined side effects (like neurotoxicity) [21]. Upon further stratification by race, we found that the relationship between PWE and alcohol use only held true among the Black population and not the White population, even after adjustment for confounding variables. In a previous retrospective study, researchers found that African American patients with temporal lobe epilepsy were more likely to have normal MRIs, to have seizure onset in adulthood, and to be female [23]. This study underscores the racial disparities in the presentation of epilepsy, which need to be taken into consideration. In our study, we found ethnic disparities in the relationship between alcohol, epilepsy, and mortality. One theory is that this is due to health disparities and social determinants of health [24]. Johnson et al., from a meta-analysis comprising 13 studies, found that Black and Hispanic Medicaid recipients were less likely to receive newer antiseizure medications [25].

Understanding the relationship between the social determinants of health (SDoH) and individuals with epilepsy provides a unique lens and opportunity that can be leveraged to facilitate care options. Equally important is the necessity to understand the extent to which various SDoH elements affect individuals impacted by epilepsy. Gaps in these areas were highlighted in research conducted by Kusyk et al. [26,27,28]. In this research, it was noted that prior research efforts focused on the Social Vulnerability Index (SVI) and the socioeconomic status measures (SES). However, the vast amount of data associated with patient-specific SDoH was not as prevalent until recently.

Further review of the research conducted by Kusyk (2025) revealed that multiple SDoH domains were covered, and the designed methodological survey was administered to patients in both inpatient and outpatient categories [28]. Of the 2984 patients meeting the inclusion criteria, SDoH need was apparent in a significant number of metrics in univariate regression. While patients with SDoH needs reported more outpatient visits, they did not report more office visits. The study results identified the benefit of using a patient-reported assessment tool, and higher SDoH needs were associated with higher healthcare utilization.

In other molecular research, targeted gene panels were used, which consist of sets of genes that have been causally implicated in a particular phenotype [23,29,30,31,32,33,34]. In contrast to our findings, another study had shown that among PWE, the diagnostic yield of genomic sequencing was significantly greater than that of targeted gene panels testing in self-reported Hispanic/Latino(a) (17.2% vs. 9.5%) and White/European American (19.8% vs. 7.9%) population groups but not in the self-reported Black/African American (11.5% vs. 7.7%) group [35,36,37,38]. In previous research, 43 foreign-born patients with drug-resistant epilepsy showed a significant association with epilepsy. There were differences according to ethnicity. In the aforementioned study, ethnicity significantly affected the following: the verbal overall score (cognitive performance) [34], Verbal Fluency (language deficits) [32], Naming (comprehensive assessment of learning), Token Test (auditory comprehension), Digit Span, Attentional Matrices, Trail Making Test, Line Orientation Test, and Raven matrices.

Additionally, we found that there may be a direct connection between PWE and alcohol use. In a meta-analysis, previous researchers found a strong and consistent association between alcohol use and increased epilepsy risk, with a pooled relative risk exceeding twofold compared with nondrinkers. A clear dose–response relationship was observed, showing progressively higher risk with increased daily alcohol intake [39,40,41,42,43]. The study identifies heavy alcohol consumption as a significant and modifiable risk factor for epilepsy. At a molecular level, alcohol acts as a central nervous system depressant, primarily through potentiation of γ-aminobutyric acid (GABA)–mediated inhibition and suppression of glutamatergic N-methyl-D-aspartate (NMDA) receptor activity [44]. Chronic exposure, however, leads to adaptive down-regulation of GABA-A receptors and up-regulation of NMDA receptors, creating a hyperexcitable neural state upon cessation of alcohol use [45]. This imbalance predisposes individuals to withdrawal seizures, typically occurring within 6–48 h after the last drink [45,46,47]. Some biological correlates can be inferred from our epidemiological findings.

Clinically, differentiation between alcohol withdrawal seizures and intrinsic epilepsy is vital. In an observational study, researchers assessed alcohol use and alcohol-related seizures among adults with epilepsy. Although approximately two-thirds of participants reported alcohol consumption, alcohol-related seizures were exclusively associated with episodes of acute heavy drinking. Individuals with generalized genetic epilepsy and chronic heavy alcohol use demonstrated significantly higher seizure risk. Light or moderate alcohol intake was not associated with increased seizure frequency, suggesting that seizure exacerbation is primarily linked to binge drinking rather than moderate consumption. Morphologically, withdrawal seizures occur within 6–48 h, lacking a focal onset; laboratory workup using EEG and imaging identifies persistent epileptiform activity. Alcohol negatively interacts with antiseizure medications such as phenytoin and valproate, necessitating cautious pharmacologic management [39,40]. On a biological level, neuronal excitability in the hippocampus was shown to be modulated by activation of this receptor, which is also linked to microglia activation and neuro-inflammatory reactions, neuronal hyperexcitability, and increased ATP [21].

### 4.1. Diagnostic Challenges

Differentiating alcohol withdrawal seizures from primary epileptic seizures is crucial for appropriate management. Withdrawal seizures typically occur within 48 h of abstinence and often lack focal onset—which was beyond the scope of this study [3]. Electroencephalography (EEG) abnormalities may persist beyond withdrawal, necessitating follow-up to rule out underlying epilepsy [3]. Alcohol interferes with the pharmacokinetics of multiple antiseizure medications (ASMs), including phenytoin, carbamazepine, and valproate, potentially leading to subtherapeutic levels or hepatotoxicity [45]. Moreover, overlapping sedative effects increase fall and injury risk. Clinicians should select ASMs with favorable hepatic profiles and provide close monitoring in patients who continue to drink. Future studies should also include the interaction between alcohol and epilepsy type (i.e., juvenile myoclonic epilepsy, temporal lobe epilepsy, and others). Genetic and epigenetic studies should also include phenotypes that are directly linked to epilepsy. Despite these limitations, this study extends prior work by leveraging longitudinal mortality linkage, multi-year national data, and stratified survival analyses. The findings underscore the importance of routine alcohol-use screening and integrated care approaches for individuals with epilepsy, particularly in populations experiencing disproportionate health risks, while emphasizing the need for prospective research to clarify causal pathways. Also, various researchers have identified the necessity to reduce the SES gap and its impact on individuals with a diagnosis of epilepsy. Additionally, the findings from our research necessitate the identification of socioeconomic disparities relative to managing the care of individuals suffering from epilepsy.

### 4.2. Recommendations

Benzodiazepines such as diazepam or lorazepam remain first-line agents for alcohol withdrawal and prevention of withdrawal seizures [45,47]. Adjunctive agents such as carbamazepine or valproate may be beneficial in patients with pre-existing epilepsy [48]. Medically supervised detoxification is strongly recommended to prevent status epilepticus and relapse.

Comprehensive management should address both disorders concurrently. Psychosocial interventions—including cognitive-behavioral therapy (CBT), motivational interviewing, and relapse prevention programs—improve adherence and seizure control [48,49]. Collaboration between neurologists, addiction specialists, and mental health providers can enhance outcomes and reduce recurrence risk.

PWE should receive individualized counseling regarding alcohol use. Small or moderate consumption may not trigger seizures in well-controlled cases, but heavy episodic drinking or abrupt cessation poses a significant risk [4]. Regular follow-up, liver function monitoring, and therapeutic drug level assessment are recommended. For alcohol-dependent individuals, pharmacotherapies such as naltrexone or acamprosate can be considered, though interactions with ASMs require evaluation [47].

### 4.3. Limitations

There are multiple limitations that highlight gaps in the diagnosis of epilepsy. Due to the observational nature of this study, more longitudinal studies are needed to establish causation, as it is unclear which comes first—alcohol use or epilepsy. Therefore, prospective cohort studies after controlling for confounders are needed to clarify whether alcohol independently causes epilepsy or acts via secondary mechanisms. Also, complementing observational studies, there should be neuroimaging biomarkers through advanced MRI and PET studies. This may help identify alcohol-related network changes that predispose individuals to epilepsy. Additionally, in our observational study, there was a potential for social desirability bias. This definition does not account for epilepsy type, severity, or diagnostic confirmation. Also, epilepsy status is based on self-reported physician diagnosis, which may introduce recall bias and misclassification as well. Also, there should be further stratification based on the severity of alcohol use and epilepsy. Combining former and current alcohol users may introduce bias, particularly due to the “sick quitter” effect. Former drinkers often have higher baseline health risks. Our approach of dichotomizing may oversimplify patterns of drinking. There is a need to distinguish between light, moderate, heavy, or binge drinking patterns by conducting future primary studies. Finally, antiseizure medication use, epilepsy severity, and duration were not collected in the NHIS dataset.

### 4.4. Future Directions

There are a number of areas in which future research is appropriate based on this study. These future directions are the following:

1. Integrated Interventions: Controlled trials testing combined neurology–addiction programs could guide best practices for dual-diagnosis management.
2. SUDEP and Mortality: Further investigation into alcohol’s contribution to SUDEP and overall epilepsy mortality is warranted.
3. During primary care visits, healthcare providers who treat seizures should also ask screening questions (Cut Down, Annoyed, Guilty, Eye-Opener—CAGE).
4. At a biomolecular level, research should address increasing the 21 ILAE (International League Against Epilepsy)-recommended genes [1,2].

## 5. Conclusions

Alcohol misuse represents a preventable, modifiable risk factor for seizures and poor epilepsy outcomes. In this nationally representative cohort, the combined presence of epilepsy and alcohol use was associated with higher all-cause mortality compared with alcohol use alone. These findings should be interpreted as hypothesis-generating rather than causal. They support the importance of alcohol-use screening and integrated clinical management among PWE and highlight the need for prospective studies to clarify causal pathways and address potential disparities in outcomes [49,50,51,52,53,54,55]. Clinicians must maintain vigilance in screening for alcohol use among individuals with epilepsy and offer coordinated, evidence-based care addressing both disorders. Interdisciplinary research and treatment integration hold promise for reducing the global burden of epilepsy and alcohol-related morbidity.

## Figures and Tables

**Table 1:** Characteristics of study participants stratified by epilepsy.

Characteristics	Total Population (*n* = 81,750)	Epilepsy (+) (*n* = 1430)	Epilepsy (−) (*n* = 80,320)
Alcohol Use **			
Lifetime Abstainer	28.4 (27.4–29.4)	31.4 (25.0–37.8)	28.3 (27.3–29.3)
Former Drinker	17.3 (16.4–18.2)	26.4 (19.9–32.8)	17.1 (16.2–18.0)
Current Drinker	54.3 (53.2–55.5)	42.3 (35.2–49.3)	54.6 (53.4–55.8)
Myocardial Infarction (MI) **	3.8 (3.6–3.9)	7.3 (5.8–9.2)	3.7 (3.6–3.9)
Stroke **	3.2 (3.1–3.4)	15.2 (13.3–17.1)	3.0 (2.9–3.1)
Chronic Kidney Disease (CKD) **	2.0 (1.9–2.1)	5.7 (4.6–7.10)	1.9 (1.8–2.0)
Diabetes **	9.7 (9.5–9.9)	12.9 (10.8–15.0)	9.6 (9.4–9.9)
Obesity **	27.8 (27.4–28.2)	32.1 (29.3–34.9)	27.7 (27.2–28.1)
Smoking Status **	42.7 (42.2–43.3)	55.5 (52.6–58.4)	42.5 (42.0–43.0)
Age (SE) **	48.3 (0.15)	47.5 (0.47)	48.3 (0.15)
**Gender Male**	45.4 (44.9–45.8)	44.2 (41.1–47.3)	45.4 (44.9–45.9)
**Family Poverty–Income Ratio** (PIR < 2) **	35.9 (35.1–36.7)	57.4 (54.0–60.8)	35.5 (34.7–36.3)
Ethnicity **			
Non-Hispanic White	71.2 (70.5–71.9)	76.9 (74.7–79.1)	71.1 (70.4–71.8)
Non-Hispanic Black	12.4 (11.9–12.9)	14.0 (12.2–15.9)	12.4 (11.8–12.9)
Hispanic	12.1 (11.7–12.5)	8.0 (6.7–9.4)	12.2 (11.7–12.6)
Other	4.3 (4.1–4.5)	1.2 (0.7–1.8)	4.3 (4.1–4.6)
Education Level **			
Some High School	14.1 (13.7–14.5)	20.6 (18.2–22.9)	14.0 (13.6–14.4)
High School Graduate	26.4 (25.9–26.9)	30.5 (27.6–33.4)	26.3 (25.8–26.8)
Some College	30.9 (30.4–31.4)	30.9 (28.1–33.6)	30.9 (30.4–31.4)
College Graduate and Above	28.6 (27.9–29.2)	18.1 (15.7–20.4)	28.8 (29.1–29.4)

Note. ** *p* < 0.01. Numbers with 95% CI indicate 95% confidence intervals for proportions.

**Table 2:** Risk of all-cause mortality among White adults 18 years or older having epilepsy and alcohol consumption: NHIS 2008–2018.

	Total Population HR (95% CI)	Epilepsy- Alcohol Use+ HR (95% CI)	Epilepsy+ Alcohol Use- HR (95% CI)	Epilepsy+ Alcohol Use+ HR (95% CI)
**Epilepsy/Alcohol**	1.01 (0.82–1.23)	0.91 (0.84–0.98) *	0.91 (0.71–1.17)	1.17 (0.85–1.62)
**Myocardial Infarction**	1.03 (0.93–1.14)	1.03 (0.93–1.14)	1.11 (0.98–1.26)	0.93 (0.80–1.08)
**Stroke**	1.10 (0.97–1.24)	1.08 (0.95–1.22)	1.08 (0.92–1.27)	1.13 (0.94–1.35)
**Chronic Kidney Disease (CKD)**	1.46 (1.25–1.70) **	1.46 (1.25–1.70) **	1.32 (1.11–1.58) **	1.75 (1.38–2.22) **
**Diabetes**	0.95 (0.87–1.03)	0.93 (0.85–1.01)	0.94 (0.84–1.05)	0.96 (0.84–1.09)
**Obesity**	0.99 (0.92–1.08)	1.00 (0.92–1.09)	0.97 (0.86–1.08)	1.03 (0.92–1.15)
**Smoking Status**	1.06 (0.99–1.14)	1.07 (1.00–1.15)	1.17 (1.06–1.28) **	0.98 (0.88–1.09) **
**Age**	1.01 (1.01–1.01) **	1.01 (1.00–1.01) **	1.01 (1.01–1.02) **	1.01 (1.00–1.01) **
**Gender (Ref. Female)**	1.07 (0.99–1.15)	1.08 (1.00–1.15) *	1.11 (0.99–1.24)	1.03 (0.93–1.14)
**Family Poverty–Income Ratio** (Ref: PIR < 2)	1.12 (1.04–1.22) **	1.12 (1.03–1.21) *	1.14 (1.03–1.26) *	1.11 (0.98–1.25)
**Education Level** College Graduate or Above Some High School High School Graduate Some College	Ref 1.02 (0.91–1.14) 0.95 (0.86–1.05) 0.92 (0.83–1.02)	Ref 1.01 (0.90–1.13) 0.94 (0.84–1.034) 0.91 (0.82–1.01)	Ref 0.92 (0.78–1.09) 0.90 (0.76–1.06) 0.94 (0.79–1.12)	Ref 1.14 (0.95–1.36) 1.01 (0.88–1.15) 0.90 (0.79–1.02)

Note. * *p* < 0.05; ** *p* < 0.01. HR (95% CI) indicates hazard ratios with 95% confidence intervals for the outcome (i.e., mortality). Ref indicates the reference group among each variable for comparison with other groups.

**Table 3:** Risk of all-cause mortality among Black adults 18 years or older having epilepsy with alcohol consumption: NHIS 2008–2018.

	Total Population HR (95% CI)	Epilepsy- High Alcohol Use + HR (95% CI)	Epilepsy+ Alcohol Use- HR (95% CI)	Epilepsy+ Alcohol Use+ HR (95% CI)
**Epilepsy/Alcohol Use**	1.31 (1.02–1.68) *	0.93 (0.80–1.09)	1.09 (0.80–1.49)	2.01 (1.18–3.40) **
**Myocardial Infarction**	1.17 (0.95–1.44)	1.19 (0.96–1.47)	1.24 (0.96–1.60)	1.04 (0.74–1.46)
**Stroke**	0.93 (0.72–1.20)	0.91 (0.70–1.18)	0.94 (0.70–1.25)	0.94 (0.59–1.50)
**Chronic Kidney Disease (CKD)**	1.19 (0.96–1.46)	1.11 (0.90–1.36)	1.11 (0.88–1.40)	1.32 (0.88–1.99)
**Diabetes**	1.02 (0.85–1.22)	1.04 (0.87–1.25)	1.03 (0.83–1.29)	0.99 (0.74–1.32)
**Obese**	0.80 (0.69–0.93)*	0.80 (0.69–0.93)	0.88 (0.72–1.08)	0.72 (0.57–0.89) *
**Smoking Status**	1.13 (0.98–1.31)	1.13 (0.97–1.32)	1.22 (1.01–1.47)	1.00 (0.78–1.28)
**Age**	1.00 (1.00–1.01)	1.00 (1.00–1.01)	1.01 (1.00–1.01)	1.00 (0.99–1.01)
**Gender (Ref. Female)**	0.88 (0.77–1.02)	0.89 (0.77–1.04)	0.88 (0.73–1.06)	0.86 (0.68–1.10)
**Family Poverty–Income Ratio** (Ref: PIR < 2)	1.07 (0.91–1.26)	1.07 (0.91–1.26)	0.93 (0.76–1.15)	1.23 (0.96–1.56)
**Education Level** College Graduate or Above Some High School High School Graduate Some College	Ref 1.08 (0.86–1.35) 1.11 (0.90–1.37) 1.14 (0.88–1.48)	Ref 1.08 (0.86–1.35) 1.09 (0.89–1.34) 1.16 (0.90–1.50)	Ref 1.15 (0.83–1.59) 1.30 (0.94–1.79) 1.27 (0.90–1.79)	Ref 1.05 (0.72–1.54) 0.97 (0.70–1.35) 1.08 (0.75–1.56)

Note. * *p* < 0.05; ** *p* < 0.01. HR (95% CI) indicates hazard ratios with 95% confidence intervals for the outcome (i.e., mortality). Ref indicates the reference group among each variable for comparison with other groups.

## Data Availability

Data can be found on the following website: https://wwwn.cdc.gov/nchs/nhanes/default.aspx, accessed on 1 March 2026.

## References

[1] WHO (2023). NIAAA. https://www.paho.org/en/topics/epilepsy.

[2] Jaffer U., Rosli F.A., Rosli N.R.N., Sani Q.D.M., Nassir C.M.N.C.M., Ahmed M.A., Osman R.A.H. (2024). Alcohol use disorder and its adverse health outcomes: A narrative review on policy, clinical intervention and future directions. Int. J. Educ. Psychol. Couns..

[3] Bråthen G., Ben-Menachem E., Brodtkorb E., Galvin R., Garcia-Monco J.C., Halasz P., Hillbom M., Leone M.A., Young A.B., EFNS Task Force on Diagnosis and Treatment of Alcohol-Related Seizures (2005). EFNS guideline on the diagnosis and management of alcohol-related seizures: Report of an EFNS task force. Eur. J. Neurol..

[4] ILAE Consortium (2018). Genome-wide mega-analysis identifies 16 loci and highlights diverse biological mechanisms in the common epilepsies. Nat. Commun..

[5] Pellacani S., Dosi C., Valvo G., Moro F., Mero S., Sicca F., Santorelli F.M. (2020). Customized multigene panels in epilepsy: The best things come in small packages. Neurogenetics.

[6] Zhou Z., Metten P., Yuan Q., Sun H., Hodgkinson C.A., Shen P.H., Marietta C., Crabbe J.C., Goldman D. (2022). Genetic and genomic signatures in ethanol withdrawal seizure-prone and seizure-resistant mice implicate genes involved in epilepsy and neuronal excitability. Mol. Psychiatry.

[7] Moe J.S., Bramness J.G., Bolstad I., Mørland J.G., Gorwood P., Ramoz N. (2025). Association Between GABRG2 and Self-Rating of the Effects of Alcohol in a French Young Adult Sample. Risk Manag. Healthc. Policy.

[8] Charsouei S. (2021). Investigating Non-pharmacological Treatments for Psychological Problems Associated with Epilepsy: A Narrative Review. Health Technol. Assess. Action.

[9] Woo K.N., Kim K., Kim H.W., Kim Y.H. (2022). Alcohol consumption on unprovoked seizure and epilepsy: An up-dated meta-analysis. Drug Alcohol Depend..

[10] Fang M., Liu W., Tuo J., Liu M., Li F., Zhang L., Yu C., Xu Z. (2023). Advances in understanding the pathogenesis of post-traumatic epilepsy: A literature review. Front. Neurol..

[11] Silva-Cardoso G.K., N’Gouemo P. (2025). Intermittent short-and long-term alcohol exposures influence alcohol consumption, anxiety-like behaviors, and seizure onset in genetically epilepsy-prone rats. Alcohol.

[12] Stawińska-Witoszyńska B., Czechowska K., Więckowska B. (2019). The prevalence of Epilepsy and its co-occurrence with alcohol dependence among polish prisoners. Int. J. Equity Health.

[13] Mayo S., Gomez-Manjon I., Marco-Hernandez A.V., Fernandez-Martinez F.J., Camacho A., Martinez F. (2023). N-type Ca channel in epileptic syndromes and epilepsy: A systematic review of its genetic variants. Int. J. Mol. Sci..

[14] Lhatoo S.D., Faulkner H.J., Dembny K., Trippick K., Johnson C., Bird J.M. (2010). An electroclinical case-control study of sudden unexpected death in epilepsy. Ann. Neurol..

[15] Hussain Z. (2024). Sodium channel hydrogen bonding in epilepsy: Molecular physiology and biophysics. Int. J. Biol. Biotechnol..

[16] Kwon C.S., Rafati A., Ottman R., Christensen J., Kanner A.M., Jetté N., Newton C.R. (2025). Psychiatric Comorbidities in Persons with Epilepsy Compared with Persons Without Epilepsy: A Systematic Review and Meta-Analysis. JAMA Neurol..

[17] Fiest K.M., Dykeman J., Patten S.B., Wiebe S., Kaplan G.G., Maxwell C.J., Bulloch A.G., Jette N. (2013). Depression in epilepsy: A systematic review and meta-analysis. Neurology.

[18] Luu K.Y., Zhao M., Mannis M.J. (2020). The use of electrotherapeutics in oph-thalmology. Am. J. Ophthalmol..

[19] Chen L., Wang Y., Chen Z. (2020). Adult neurogenesis in epileptogenesis: An up-date for preclinical finding and potential clinical translation. Curr. Neuropharmacol..

[20] Baculis B.C., Weiss A.C., Pang W., Jeong H.G., Lee J.H., Liu D.C., Tsai N.P., Chung H.J. (2017). Prolonged seizure activity causes caspase dependent cleavage and dysfunction of G-protein activated inwardly rectifying potassium channels. Sci. Rep..

[21] Sumadewi K.T., Harkitasari S., Tjandra D.C. (2023). Biomolecular mechanisms of epileptic seizures and epilepsy: A review. Acta Epileptol..

[22] Ribierre T., Baulac S. (2025). Du mosaïcisme cérébral aux crises d’épilepsie—Une nouvelle stratégie thérapeutique ciblée [From brain mosaicism to epilepsy: Toward a novel targeted therapeutic strategy]. Med. Sci..

[23] Ravizza T., Scheper M., Di Sapia R., Gorter J., Aronica E., Vezzani A. (2024). mTOR and neuroinflammation in epilepsy: Implications for disease progression and treatment. Nat. Rev. Neurosci..

[24] Phoswa W.N., Mokgalaboni K. (2023). Immunological imbalances associated with epileptic seizures in type 2 diabetes mellitus. Brain Sci..

[25] Allen S.E., Limdi N.A., Westrick A.C., Ver Hoef L.W., Szaflarski J.P., Kuzniecky R.I., Knowlton R.C. (2019). Racial differences in adult-onset MRI-negative temporal lobe epilepsy. Epilepsy Behav..

[26] Reyes A., Prabhakaran D., Banegas M.P., Shih J.J., Iragui-Madoz V.J., Almane D.N., Ferguson L., Jones J.E., Busch R.M., Hermann B.P. (2024). Individual-and community-level social determinants of health are associated with cognition in older adults with focal epilepsy. Epilepsy Behav..

[27] Johnson B.J., Jung K.E., MacKenzie M.A., Bah A., Jetté N., Mohamed N., Blank L.J. (2025). Association of social determinants of health with first antiseizure medication prescription for patients with newly diagnosed epilepsy: A systematic review and meta-analysis. Epilepsia.

[28] Kusyk D.M., Jaffee S., Lejeune K., Brignone E., Yin Y., Li J., Whiting A.C. (2025). The effects of social determinants of health on patients with epilepsy. Epilepsy Behav..

[29] Abrego A.M., Khan W., Wright C.E., Islam M.R., Ghajar M.H., Bai X., Tandon N., Seymour J.P. (2023). Sensing local field potentials with a directional and scalable depth electrode array. J. Neural Eng..

[30] Lauerer R.J., Lerche H. (2024). Voltage-gated calcium channels in genetic epilepsies. J. Neurochem..

[31] Okoh J., Mays J., Bacq A., Oses-Prieto J.A., Tyanova S., Chen C.J., Imanbeyev K., Doladilhe M., Zhou H., Jafar-Nejad P. (2023). Targeted suppression of mTORC2 reduces seizures across models of epilepsy. Nat. Commun..

[32] Proietti Onori M., Koene L.M.C., Schäfer C.B., Nellist M., de Brito van Velze M., Gao Z., Elgersma Y., van Woerden G.M. (2021). RHEB/mTOR hyperactivity causes cortical malformations and epileptic seizures through increased axonal connectivity. PLoS Biol..

[33] Huang X.Y., Hu Q.P., Shi H.Y., Zheng Y.Y., Hu R.R., Guo Q. (2021). Everolimus inhibits PI3K/Akt/mTOR and NF-kB/IL-6 signaling and protects seizure-induced brain injury in rats. J. Chem. Neuroanat..

[34] Al-Kuraishy H.M., Jabir M.S., Al-Gareeb A.I., Albuhadily A.K., Klionsky D.J., Rafeeq M.F. (2025). Epilepsy and autophagy modulators: A therapeutic split. Autophagy.

[35] Zhang X., Wu Z., Zhou X., Tao H. (2025). Mitochondrial dysfunction in epilepsy: Mechanistic insights and clinical strategies. Mol. Biol. Rep..

[36] Świątkowski W., Smyk P., Pankowski D., Marcinkowski T. (2019). Alcohol dependence and epilepsy among prisoners in Poland. Int. J. Equity Health.

[37] Lhatoo S.D., Faulkner H.J., Duncan J.S., Sander J.W. (2020). Mortality and SUDEP in epilepsy with alcohol use disorder: A population-based study. Front. Neurol..

[38] Abul-Husn N.S., Marathe P.N., Kelly N.R., Bonini K.E., Sebastin M., Odgis J.A., Abhyankar A., Brown K., Di Biase M., Gallagher K.M. (2023). Molecular diagnostic yield of genome sequencing versus targeted gene panel testing in racially and ethnically diverse pediatric patients. Genet. Med..

[39] Reardon A.D., Gillinder L., Copland D.A., McMahon K.L., Brownsett S.L.E. (2025). Uncovering language deficits in focal epilepsy: Beyond the limits of noun naming and verbal fluency. Epilepsy Behav..

[40] Van Patten R., Austin T.A., Cotton E., Chan L., Bellone J.A., Mordecai K., Altalib H., Correia S., Twamley E.W., Jones R.N. (2024). Cognitive performance in functional seizures compared with epilepsy and healthy controls: A systematic review and meta analysis. Lancet Psychiatry.

[41] Puntambekar I., Koepp M., Xiao F., Baxendale S. (2025). A systematic survey of the measures used to identify postoperative changes in language function following epilepsy surgery. Epilepsia Open.

[42] Peviani V., Scarpa P., Toraldo A., Bottini G. (2016). Accounting for ethnic-cultural and linguistic diversity in neuropsychological assessment of patients with drug-resistant epilepsy: A retrospective study. Epilepsy Behav..

[43] Zhao J., Stockwell T., Naimi T., Churchill S., Clay J., Sherk A. (2023). Association between daily alcohol intake and risk of all-cause mortality: A systematic review and meta-analyses. JAMA Netw. Open.

[44] Quelch D., Lingford-Hughes A., John B., Nutt D., Bradberry S., Roderique-Davies G. (2025). Promising strategies for the prevention of alcohol-related brain damage through optimised management of acute alcohol withdrawal: A focussed literature review. J. Psychopharmacol..

[45] Masicampo M.L., Shan H.Q., Xu V., Speagle M., Godwin D.W. (2018). Selective blockade of T-type Ca2+ channels is protective against alcohol-withdrawal induced seizure and mortality. Alcohol Alcohol..

[46] Bisschop J.M., de Jonge H.J., Brunsveld-Reinders A.H., van de Mheen D.H., Mathijssen J.J., Rozema A.D. (2025). Screening instruments to detect problematic alcohol use among adults in hospitals and their diagnostic test accuracy: A systematic review. Drug. Alcohol Rev..

[47] Anand S.K., Ahmad M.H., Sahu M.R., Subba R., Mondal A.C. (2023). Detrimental effects of alcohol-induced inflammation on brain health: From neurogenesis to neurodegeneration. Cell. Mol. Neurobiol..

[48] Soyka M. (2017). Treatment of benzodiazepine dependence. N. Engl. J. Med..

[49] Ernawati I., Islamiyah W.R. (2018). How to improve clinical outcome of epileptic seizure control based on medication adherence? A literature review. Open Access Maced. J. Med. Sci..

[50] Supriya S., Jan T., Sidnal N., Thompson-Whiteside S. (2022). Alcoholic EEG data classification using weighted graph-based technique. International Conference on Health Information Science.

[51] Chahal C.A.A., Gottwald J.A., St Louis E.K., Xie J., Brady P.A., Alhurani R.E., Timm P., Thapa P., Mandrekar J., So E.L. (2022). QT prolongation in patients with index evaluation for seizure or epilepsy is predictive of all-cause mortality. Heart Rhythm.

[52] Moon H.J., Lee H., Yoon D., Koo Y.S., Shin J.Y., Lee S.Y. (2023). Premature mortality and causes of death among people with epilepsy: A nationwide population-based incident cohort study. Neurology.

[53] Dreier J.W., Laursen T.M., Tomson T., Plana-Ripoll O., Christensen J. (2023). Cause-specific mortality and life years lost in people with epilepsy: A Danish cohort study. Brain.

[54] Rheims S., Sperling M.R., Ryvlin P. (2022). Drug-resistant epilepsy and mortality—Why and when do neuromodulation and epilepsy surgery reduce overall mortality. Epilepsia.

[55] Shah R.A., Chahal C.A.A., Ranjha S., Dabbagh G.S., Asatryan B., Limongelli I., Khanji M., Ricci F., De Paoli F., Zucca S. (2024). Cardiovascular disease burden, mortality, and sudden death risk in epilepsy: A UK Biobank study. Can. J. Cardiol..

